# The ALA5/ALA6/ALA7 repeat polymorphisms of the *glutathione peroxidase‐1* ( *GPx1* ) gene and autism spectrum disorder

**DOI:** 10.1002/aur.2655

**Published:** 2022-01-08

**Authors:** Federica Carducci, Chiara Ardiccioni, Rosamaria Fiorini, Arianna Vignini, Alice Di Paolo, Sonila Alia, Marco Barucca, Maria Assunta Biscotti

**Affiliations:** ^1^ Dipartimento di Scienze Della Vita e Dell'Ambiente Università Politecnica Delle Marche Ancona Italy; ^2^ New York‐Marche Structural Biology Center (NY‐MaSBiC) Università Politecnica Delle Marche Ancona Italy; ^3^ Dipartimento di Scienze Cliniche Specialistiche ed Odontostomatologiche Università Politecnica Delle Marche Ancona Italy

**Keywords:** ASD, autism spectrum disorder, glutathione peroxidase 1, GPx1 genetic screening, GPx1 in vitro protein production, GPx1 polymorphisms, GPx1 protein activity

## Abstract

Autism is a severe neurodevelopmental disorder leading to deficits in social interaction, communication, and several activities. An increasing number of evidence suggests a role of oxidative stress in the etiology of autism spectrum disorder (ASD). Indeed, impaired antioxidant mechanisms may lead to the inadequate removal of H_2_O_2_ with a consequent increase in highly active hydroxyl radicals and other reactive oxygen species causing cellular damages. The GPx1 is one of the most important enzymes counteracting oxidative stress. In this work, we investigated a possible correlation between the GCG repeat polymorphism present in the first exon of *GPx1* gene encoding a tract of five to seven alanine residues (ALA5, ALA6, and ALA7) and ASD. Our findings highlighted a high frequency of ALA5 allele in ASD subjects. Moreover, proteins corresponding to the three GPx1 variants were produced in vitro*,* and the evaluation of their activity showed a lower values for GPx1 having ALA5 polymorphism. The comparison of the secondary and tertiary structure predictions revealed an alpha‐helix in correspondence of alanine stretch only in the case of GPx1‐ALA7 variant. Finally, to better investigate protein structure, steady‐state fluorescence measurements of GPx1 intrinsic tryptophan were carried out and the three tested proteins exhibited a different stability under denaturing conditions. This work demonstrates the importance in adopting a multidisciplinary strategy to comprehend the role of GPx1 in ASD.

**Lay Summary:**

Results here obtained suggest a possible role of ALA5 GPx1 variant in ASD. However, given the multifactorial nature of autism, this evidence might be a piece of a more complex puzzle being the GPx1 enzyme part of a complex pathway in which several proteins are involved.

## Lay summary

Results here obtained suggest a possible role of ALA5 GPx1 variant in ASD. However, given the multifactorial nature of autism, this evidence might be a piece of a more complex puzzle being the GPx1 enzyme part of a complex pathway in which several proteins are involved.

## INTRODUCTION

Autism is a severe neurodevelopmental disorder (Lord et al., 2001; Yeargin‐Allsopp et al., 2003) and individuals affected are characterized by deficits in social interaction, communication, and several activities. The etiology of the autism has not been defined given also to the heterogeneity of this disorder. However, an increasing number of evidence suggests a role for oxidative stress. The production of reactive oxygen species (ROS) is counteracted by the antioxidant capacity of the cell in normal subjects. When this equilibrium is missing, the increasing of ROS levels may determine a reduction of brain cell number and thus resulting in autistic pathology (Ghanizadeh et al., 2012). Glutathione peroxidases (GSH‐Px) are among the major enzymes for defense against oxidant molecules. These enzymes belong to a family consisting of eight genes encoding isozymes having different location and substrate specificity. GPx1 is a soluble selenoprotein that reduces H_2_O_2_ and organic hydroperoxides to water by using reduced glutathione (GSH) and reduced NADPH as cofactors. This enzyme is a homotetramer in which each subunit of 22 kDa contains a selenocysteine residue at the amino acid position 47. GPx1 is the most abundant and ubiquitous among the GPx isozymes and is mainly found in the cytoplasm. At genomic level, the *GPx1* gene is located on chromosome 3p21.3 and contains two exons (Kiss et al., 1997).

Genetic and biochemical studies have investigated the protein activity and nucleotide mutations in *GPx1* in ASD. A study performed on 30 Saudi autistic children (22 males and 8 females) aged 3–15 years and 30 healthy children, as control group, has evaluated the oxidative stress and antioxidant‐related parameters in plasma and red blood cells. The enzymatic activity of GSH‐Px was higher in autistic children compared to controls (Al‐Gadani et al., 2009). On the contrary, a lower activity of this enzyme was reported by Ghanizadeh (2012), Meguid et al. (2011), and Yorbik et al. (2002). Abnormalities in the activity of blood antioxidant enzyme systems have been correlated with an accumulation of free radicals that could damage brain tissue. Söğüt et al. (2003) have found increased GSH‐Px activity in plasma of autistic patients compared to controls. The behavior of GSH‐Px has been attributed to the increase in lipid peroxidation and overproduction of H_2_O_2_.

Concerning genetic studies on *GPx1* gene, Ming et al. (2010) have analyzed a possible correlation between the GCG repeat polymorphism in the first exon coding for a polyalanine tract of five to seven alanine residues (ALA5, ALA6, and ALA7) and autism disorder. In particular, a significant under transmission of the ALA6 allele studying 103 family trios was evidenced suggesting a protective effect of this variant for ASD.

We have expanded this genetic screening analyzing data deposited in MSSNG database belonging to over 5000 affected subjects. Moreover functional proteins related to the three GPx1 variants were produced in vitro and their activity was evaluated.

## METHODS

### Genetic analyses

In the framework of a project financed by Polytechnic University of Marche, a preliminary for the ALA5/6/7 polymorphisms of the *GPx1* gene was conducted on 20 children with ASD (14 males and 6 females) in age from 5 to 10 years. The control population consists of 20 healthy age and gender‐matched controls (12 males and 8 females), visiting the hospital for routine check‐ups; all controls were in normal condition with no associated diseases. The diagnosis of autism was made by the child neuropsychiatry of Azienda Ospedaliero Universitaria, Ospedali Riuniti di Ancona, Presidio Salesi (Ancona, Italy) based on the criteria of autistic disorder as defined by ADOS protocol (Autism Disgnostic Observation Schedule). Concerning ASD subjects, children affected by multiple pathologies were not enrolled. This study was approved by ethical committee of Italian Marche Region (CERM) (Prot. 2019 372). Informed consent was obtained from the parents of both patients and healthy subjects. The study was performed in accord with the principles of the Declaration of Helsinki, as revised in 2001.

Blood samples (about 10 ml) were collected from subjects of both groups in tubes containing EDTA as anticoagulant. Total RNA was extracted using TRIzol reagent (Invitrogen) and first strand cDNA was obtained with reverse transcription using SuperScript III First Strand Reaction Mix (Invitrogen) according manufacturer's instructions. cDNA was amplified using Platinum Taq DNA Polymerase (Invitrogen). Forward 5′‐TTCCGGCTTAGGAGGAGCACGC‐3′ and reverse 5′‐AGAATGTGGCGTCCCTCTGA‐3′ primers were designed to amplify a cDNA fragment of about 381 bp at the 5′ end of *GPx1* gene. The amplified products were purified and sequenced.

To expand the number of probands the MSSNG database (https://research.mss.ng/) was used (application number DACO‐2021‐06 approved on July 14, 2021). In particular, allele and genotype frequencies were determined for 5102 affected subjects (1028 females and 4074 males) and 6079 unaffected family members (3046 females and 3033 males) for the three *GPx1* variants, ALA5, ALA6, and ALA7. Data were retrieved using the Small Variant Queries browser provided by the MSSNG database. Starting from the TSV related files downloaded, the allele lengths were determined taking into account information present in the columns “*reference allele*,” “*alternate allele*,” and “*genotype*” in the genomic region of interest. Subjects having alleles with a number of GCG repeats higher than 7 in 1000 Genome Project database present a very low frequency (e.g., it was 0.00007 for ALA8) and therefore they were not found in our dataset. Moreover, a transmission/disequilibrium test (TDT) to multi‐allelic loci was conducted to compare counts of transmitted and nontransmitted alleles from parents to offspring. Trios (1103) with all members genotyped and heterozygous parents were considered in the analysis. The significance of the observed TDT values was assessed through chi‐square test. The same analysis was also performed considering 34 family trios with an unaffected children.

### GPx1 production, purification, and activity

The plasmid pSEC‐UAG‐Evol2 was provided by Prof. Söll of University of Yale (materials transfer agreement MTO. 22,014) and used to produce the GPx1 protein containing ALA7 polymorphism. The constructs for GPx1 having ALA5 and ALA6 polymorphisms were obtained from this construct using Gibson Assembly Cloning Kit (New England BioLabs). To verify the presence of a correct CDS corresponding to the GPx1 variants of interest, the constructs were sequenced. The production of proteins related to the three GPx1 variants was performed at New York‐Marche Structural Biology Center (NY‐MaSBiC) following the protocol described by Mukai et al., 2018. The secondary and tertiary structures of the produced GPx1 proteins were predicted with I‐Tasser (Yang et al., 2015).

Their activity was followed spectrophotometrically at 340 nm and calculated from the rate of NADPH oxidation using the Glutathione Peroxidase Cellular Activity Assay Kit (Sigma).

### Steady‐state fluorescence measurements

Steady‐state fluorescence measurements of GPx1 intrinsic tryptophan (Trp) were performed to obtain informations about protein structure using a Perkin‐Elmer LS 55 and an excitation wavelength of 295 nm. When a Trp is completely exposed to the hydrophilic environment the emission maximum is about 350 nm (as for free Trp in water), while it is blue‐shifted in a very hydrophobic environment (Lakowicz, 2006). Thus, the fluorescence emission maximum gives information of the polarity of the microenvironment where Trp is located. In our analyses, final protein concentration was 120 μg/ml, in 20 mM Tris/HCl, 300 mM NaCl and 10% of glycine pH 8.5. Data were acquired at 25 and 53°C, in the presence and in the absence of 5 M urea. Samples were equilibrated at the temperature used for 10 min before data acquisition. The buffer alone showed no fluorescence.

## RESULTS AND DISCUSSION

The GPx1 is one of the most important antioxidant enzymes counteracting oxidative stress (Rotruck et al., 1973). Impaired antioxidant mechanisms may lead to the inadequate removal of H_2_O_2_ with a consequent increase in highly active hydroxyl radicals and other ROS. The presence of these molecules can cause cell membrane damage, changes in membrane fluidity and permeability, DNA and protein damage, leading to cell death through apoptosis or necrosis (Yorbik et al., 2002). Several studies proposed that impaired activities of antioxidant system might be involved in the pathophysiological role in ASD and other psychiatric diseases as schizophrenia and bipolar disorders (Akarsu et al., 2018; Fung & Hardan, 2019; Söğüt et al., 2003).

One of the most studied polymorphisms of *GPx1* gene is the GCG repeat leading to a five (ALA5), six (ALA6), and seven (ALA7) alanine residues (Winter et al., 2003). A protective role in ASD has been proposed for the ALA6 allele by Ming et al. (2010).

To better investigate the role of these variants in ASD we have undertaken a multidisciplinary approach in the framework of a project financed by Polytechnic University of Marche. A preliminary search for the ALA5/6/7 polymorphisms of the *GPx1* gene was conducted in the enrolled ASD subjects and controls. The allele frequencies did not evidence marked differences between affected individuals and controls. It is noteworthy that ASD subjects showed a higher genotype frequency of the heterozygotes ALA5/7 and ALA6/7 compared to controls. However, these data were referred to a restricted number of subjects (see Table S1 in the Supplement). Therefore, to expand our genetic investigation we accessed the MSSNG database that allowed us to consider 5102 affected subjects and 6079 unaffected family members (see Table 1). The two datasets (affected and unaffected) showed differences in allele and genotype frequencies as supported by chi‐square test (see Table S2 in the Supplement). The comparison with data reported for North American populations (NHLBI) and total data reported in 1000 Genomes highlighted a higher frequency of ALA5 and a lower frequency of ALA6 both for ASD subjects and unaffected family members. Since autism is a neurodevelopmental disorder affecting males and females with a ratio of 4:1 (Lord et al., 2001; Yeargin‐Allsopp et al., 2003), we also assessed the genotype frequencies in relation to sex in both datasets (see Table S3 in the Supplement). The chi square test did not show statistically significant differences in the genotype frequencies between males and females of affected individuals (see Table S4 in the Supplement). Using data available in the MSSNG database we performed the transmission/disequilibrium test (TDT) to multi‐allelic loci considering 1103 case trios. For the transmission of the three alleles Ming et al. (2010) have observed significant differences only for ALA6 while our analysis evidenced statistically different values for all the three *GPx1* variants (see Table 2). These observations suggested that the three *GPx1* variants might be related to ASD. Performing the TDT test on unaffected family trios, no significant differences were observed (see Table S5 in the Supplement). However, this finding was referred to a restricted number of unaffected trios available in the MSSNG database.

**TABLE 1:** Allele and genotype frequencies

	Affected ASD subjects in MSSNG database		Unaffected family members in MSSNG database			Allele frequency in NHLBI exome sequencing project		
	*N* individuals	Allele frequency	*N* individuals	Allele frequency	Allele frequencies of parents reported by Ming et al. (2010)	European‐American	African‐American	Global allele frequency in 1000 genomes
ALA5	4269	0.42	5006	0.41	0.49	0.06	0.04	0.25
ALA6	2575	0.25	2995	0.25	0.30	0.39	0.31	0.41
ALA7	3360	0.33	4157	0.34	0.22	0.55	0.65	0.35

	Affected ASD subjects in MSSNG database		Unaffected family members in MSSNG database		Genotype frequency in NHLBI exome sequencing project	
	*N* individuals	Genotype frequency	*N* individuals	Genotype frequency	European‐American	African‐American
ALA6/ALA7	627	0.12	712	0.12	0.05	0.04
ALA6/ALA5	1206	0.24	1325	0.22	0.01	–
ALA7/ALA5	927	0.18	1063	0.17	0.02	0.04
ALA7/ALA7	903	0.18	1191	0.20	0.52	0.63
ALA6/ALA6	371	0.07	479	0.08	0.36	0.29
ALA5/ALA5	1068	0.21	1309	0.22	0.05	0.04

**TABLE 2:** Transmitted and not transmitted GPx1 alleles and related statistics

	Number of alleles transmitted	Number of alleles not transmitted	χ2	*p*‐value
ALA5	787	893	6.69	<0.00001
ALA6	631	708	4.43	0.035313
ALA7	788	605	24.04	<0.00001

**FIGURE 1:**
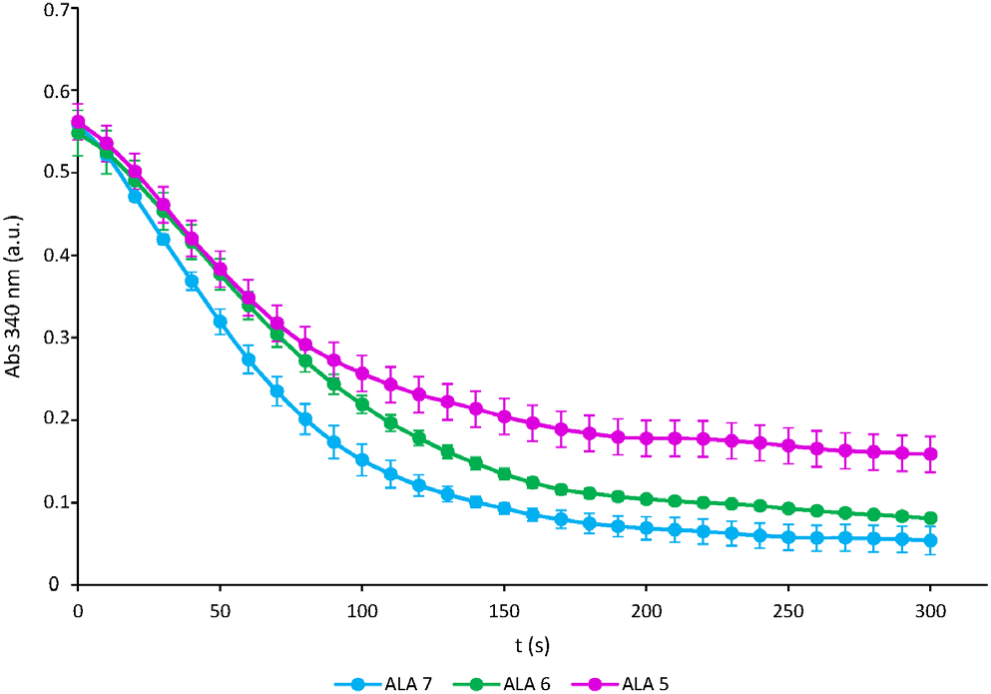
Enzyme activity of the GPx1 variants: ALA5 (purple profile), ALA6 (green profile), and ALA7 (light blue profile). The data represent the mean and SD of *n* ≥ 3 independent experiments

**FIGURE 2:**
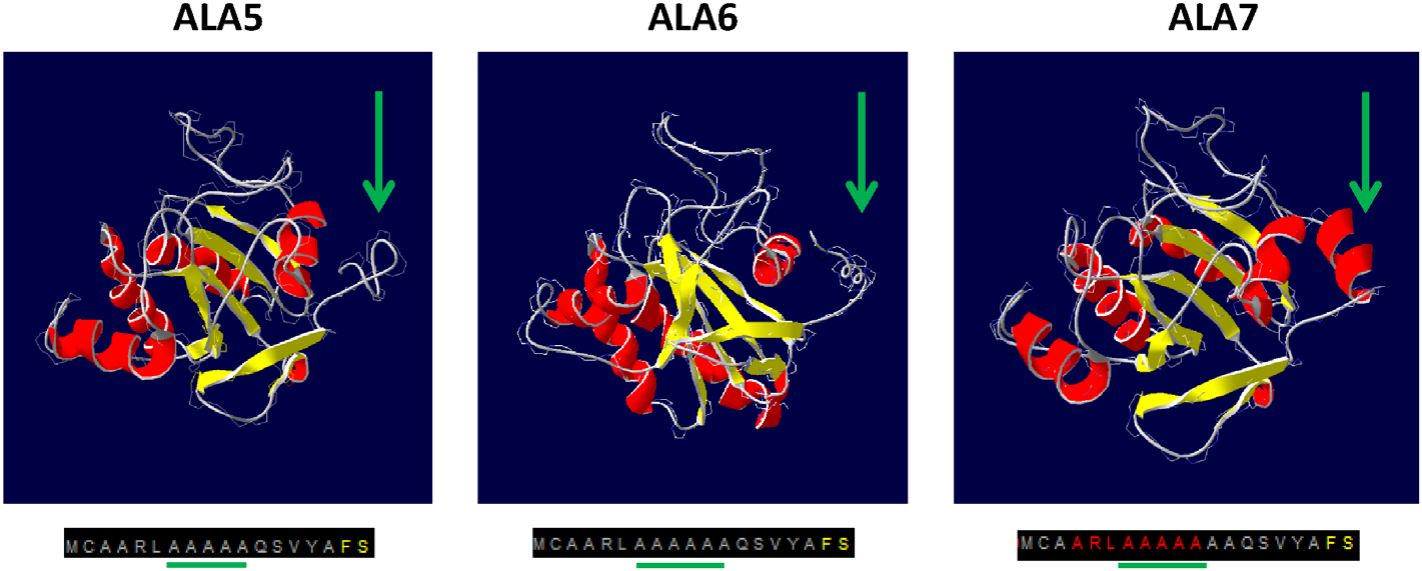
Secondary and tertiary structures prediction. The green arrows and lines indicate the region in which the alanine stretch repeat is found

**FIGURE 3:**
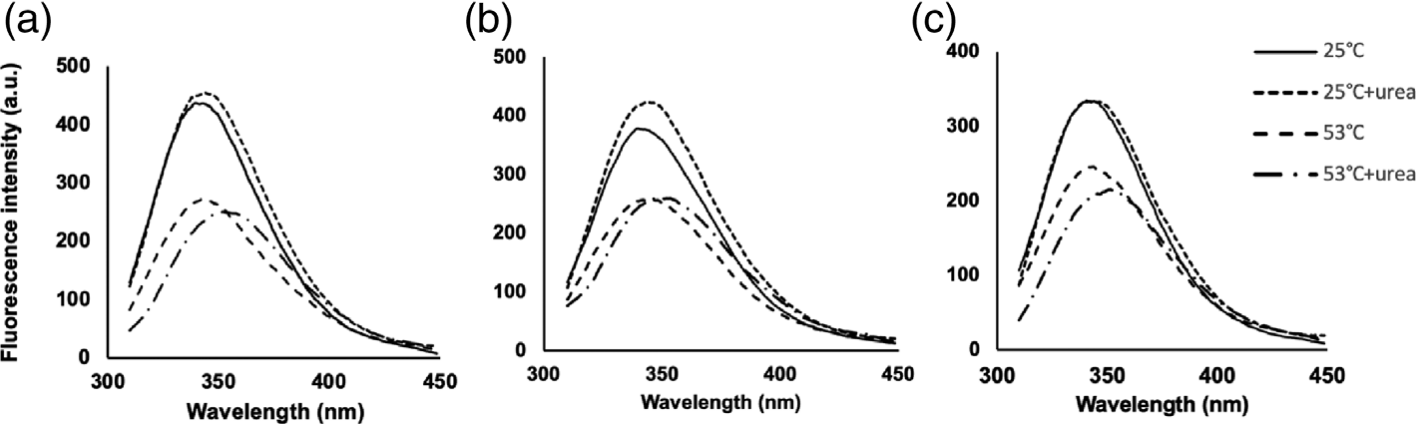
Fluorescence spectra of GPx1 ALA5 (a), ALA6 (b), and ALA7 (c) at 25°C in absence (^___^) and in presence ( ‐ ‐ ‐ ) of 5M urea; at 53°C in absence (– – –) and in presence (– ∙ – ∙ –) of 5 M urea. The excitation wavelength was set at 295 nm (bandwidth 3 nm) and the emission spectra were recorded between 310 and 450 nm (bandwidth 3.5 nm)

To better investigate the three variants, the correspondent proteins were produced in vitro following a detailed protocol (Mukai et al., 2018) to ensure the addition of the selenocysteine residue (position 49), essential for the GPx1 activity. Between the produced proteins a lower activity was detected for GPx1 having ALA5 polymorphism followed by GPx1 with ALA6 and ALA7 (see Figure 1). To understand the possible causes of differences in protein activities, the secondary and tertiary structures of the three GPx1 variants were predicted using I‐Tasser and graphically visualized through Swiss‐PDB viewer. The predictions (see Figure 2) showed that the only difference was present in the N‐ter region containing the alanine stretch. In the case of the GPx1 ALA7 variant, the prediction revealed an alpha helix structure involving eight amino acids including five alanine residues. No secondary structures were identified in the same region in the GPx1 ALA 5 and ALA 6 variants. A preliminary indication of the differences in the structure of produced proteins corresponding to the three GPx1 variants could be the different stability demonstrated under the action of urea as a denaturing agent, revealed by steady‐state fluorescence measurements of intrinsic Tryptophan (Trp) (see Figure 3). GPx1 has two Trp residues and one of them is in the catalytic site of the enzyme. Fluorescence spectra of GPx1 ALA5, ALA6, ALA7 at 25 and 53°C, in absence and presence of 5 M urea, were reported in Figure 3a–c, respectively. Trp fluorescence emission maximum has showed no significant differences in the three variants of GPx1 in all experimental conditions and it was 340 nm at 25°C, indicating a partially buried configuration of Trp. In the presence of 5 M urea at 25°C the emission maximum was slightly red shifted respect to the folded protein (344 nm vs. 340 nm) showing an increase of solvent‐exposed Trp residues; however, GPx1 ALA5 and ALA6 exhibited an increase in fluorescence intensity, compared to GPx1 ALA7, indicating a possible intramolecular quenching by some amino acids in close proximity to Trp residues. At 53°C the emission maximum was slightly red shifted respect to the folded form (344 nm vs. 340), while it was shifted to 351 nm by 5 M urea, indicating that Trp residues are totally exposed to water.

It is interesting to note that the allele ALA5 was highly frequent in ASD subjects and the activity of the GPx1 containing this variant presented lower values compared to those of other variants tested. This finding might be related to the change identified in the structural protein prediction. Therefore, this variant could play a role in autism disorder. However, the GPx1 enzyme is part of a wide pathway in which several proteins are involved and ASD has multifactorial nature and consequently the GPx1 ALA5 variant might represent a piece of a more complex puzzle. Moreover, it is also noteworthy that the GPx1 is a tetrameric protein and thus in heterozygotes, the activity of the GPx1 could be influenced by different subunits composing the tetramer. This might also explain the contrasting data reported in literature for the evaluation of the total activity of GPx obtained from blood samples (Ghanizadeh, 2012; Meguid et al., 2011; Yorbik et al., 2002).

The N‐ter region, in which alanine stretch is localized, could represent a signal sequence useful for transport and localization of GPx1 in the subcellular compartments. Therefore, the structural differences predicted at the N‐ter region could be responsible for the impaired GPx1 sorting in the cellular compartments. Bera et al. (2014) have observed a different distribution between cytoplasm and mitochondria in relation to distinct GPx1 alleles with an impact on cellular biology.

Overall, there is considerable evidence that GPx1 polymorphisms have a strong association with some diseases (Buraczynska et al., 2017; Hong et al., 2013; Jerotic et al., 2019; Souiden et al., 2016; Wei et al., 2020). In particular, the polymorphic site containing the alanine stretch has been found to be related with cancer (Jefferies et al., 2005; Kote‐Jarai et al., 2002), cardiovascular diseases (Hamanishi et al., 2004), and mental disorders such as schizophrenia (Shao et al., 2020).

Results here obtained suggest a possible role of ALA5 GPx1 variant in ASD. However, given the multifactorial nature of autism, this evidence might be a piece of a more complex puzzle being the GPx1 enzyme part of a complex pathway in which several proteins are involved. Moreover, this work demonstrates the importance in adopting a multidisciplinary strategy to provide a more holistic understanding of how genetic variants influence protein activity.

## CONFLICT OF INTEREST

The authors declare that they have no competing interests.

## Supporting information


**Table S1** Frequencies of alleles and genotypes referred to ALA5, ALA6, and ALA7 GPx1 polymorphisms in autistic subjects and controls enrolled by Ospedali Riuniti di Ancona
**Table S2** Allele and genotype frequencies observed in affected individuals and unaffected family members with related chi‐square tests and *p*‐values
**Table S3** Genotype frequencies observed in males and females of unaffected family members and affected individuals
**Table S4** Genotype frequencies observed in male and female affected individuals with related chi‐square tests and *p*‐values
**Table S5** Transmitted and not transmitted GPx1 alleles and related statistics in unaffected trios
