# Supplementary material for: The ALA5/ALA6/ALA7 repeat polymorphisms of the glutathione peroxidase‐1 ( GPx1 ) gene and autism spectrum disorder
Source: Autism Res. 2022 Jan 8;15(2):215–21. doi: 10.1002/aur.2655 (PMC9304179; doi:10.1002/aur.2655)
Supplement: Supplementary file 1 — Table S1 Frequencies of alleles and genotypes referred to ALA5, ALA6, and ALA7 GPx1 polymorphisms in autistic subjects and controls enrolled by Ospedali Riuniti di Ancona Table S2 Allele and genotype frequencies observed in affected individuals and unaffected family members with related chi‐square tests and p‐values Table S3 Genotype frequencies observed in males and females of unaffected family members and affected individuals Table S4 Genotype frequencies observed in male and female affected individuals with related chi‐square tests and p‐values Table S5 Transmitted and not transmitted GPx1 alleles and related statistics in unaffected trios [file AUR-15-215-s001.docx]

**The ALA5/ALA6/ALA7 repeat polymorphisms of the *glutathione peroxidase-1* (*GPx1*) gene and autism spectrum disorder (ASD)**

***Supplemental Information***

**Table S1.** Frequencies of alleles and genotypes referred to ALA5, ALA6, and ALA7 GPx1 polymorphisms in autistic subjects and controls enrolled by Ospedali Riuniti di Ancona.

|  | **Allele frequency**  **in ASD** | **Allele frequency**  **in controls** |
| --- | --- | --- |
| **ALA5** | 0.42 | 0.42 |
| **ALA6** | 0.42 | 0.50 |
| **ALA7** | 0.16 | 0.06 |
|  |  |  |
|  | **Heterozygous frequency in ASD** | **Heterozygous frequency in controls** |
| **ALA5/ALA6** | 0.32 | 0.39 |
| **ALA5/ALA7** | 0.21 | 0.06 |
| **ALA6/ALA7** | 0.11 | 0.06 |
|  |  |  |
|  | **Homozygous frequency in ASD** | **Homozygous frequency in controls** |
| **ALA5/ALA5** | 0.16 | 0.22 |
| **ALA6/ALA6** | 0.21 | 0.28 |
| **ALA7/ALA7** | 0 | 0 |

|  |  | **Allele frequencies** | | |  |  |
| --- | --- | --- | --- | --- | --- | --- |
|  | **Number of individuals** | **ALA5** | **ALA6** | **ALA7** | **χ2** | **p-value** |
| **ASD patients** | 5,102 | 0.42 | 0.25 | 0.33 | 7.13 | 0.028 |
| **unaffected family members** | 6,079 | 0.41 | 0.25 | 0.34 |  |  |

|  |  | **Genotype frequencies** | | | | | |  |  |
| --- | --- | --- | --- | --- | --- | --- | --- | --- | --- |
|  | **Number of individuals** | **ALA6/ALA7** | **ALA6/ALA5** | **ALA7/ALA5** | **ALA7/ALA7** | **ALA6/ALA6** | **ALA5/ALA5** | **χ2** | **p-value** |
| **ASD patients** | 5,102 | 0.12 | 0.24 | 0.18 | 0.18 | 0.07 | 0.21 | 23.07 | 0.0003 |
| **unaffected family members** | 6,079 | 0.12 | 0.22 | 0.17 | 0.20 | 0.08 | 0.22 |  |  |

**Table S2.** Allele and genotype frequencies observed in affected individuals and unaffected family members with related chi-square tests and p-values.

**Table S3.** Genotype frequencies observed in males and females of unaffected family members and affected individuals.

|  | **unaffected family members** | | | | **ASD patients** | | | |
| --- | --- | --- | --- | --- | --- | --- | --- | --- |
|  | **Males** | | **Females** | | **Males** | | **Females** | |
|  | **N individuals** | **genotype frequencies** | **N individuals** | **genotype frequencies** | **N individuals** | **genotype frequencies** | **N individuals** | **genotype frequencies** |
| **ala6/ala7** | 379 | 0,12 | 333 | 0,11 | 494 | 0,12 | 133 | 0,13 |
| **ala6/ala5** | 666 | 0,22 | 659 | 0,22 | 987 | 0,24 | 219 | 0,21 |
| **ala7/ala5** | 512 | 0,17 | 551 | 0,18 | 743 | 0,18 | 184 | 0,18 |
| **ala7/ala7** | 601 | 0,20 | 590 | 0,19 | 703 | 0,17 | 200 | 0,19 |
| **ala6/ala6** | 239 | 0,08 | 240 | 0,08 | 303 | 0,07 | 68 | 0,07 |
| **ala5/ala5** | 636 | 0,21 | 673 | 0,22 | 844 | 0,21 | 224 | 0,22 |

**Table S4.** Genotype frequencies observed in male and female affected individuals with related chi-square tests and p-values.

|  |  | **Genotype frequencies** | | | | | |  |  |
| --- | --- | --- | --- | --- | --- | --- | --- | --- | --- |
|  | **Number of individuals** | **ALA6/ALA7** | **ALA6/ALA5** | **ALA7/ALA5** | **ALA7/ALA7** | **ALA6/ALA6** | **ALA5/ALA5** | **χ2** | **p-value** |
| **Male ASD patients** | 4,074 | 0.12 | 0.24 | 0.18 | 0.17 | 0.07 | 0.21 | 1.40 | 0.92 |
| **ASD patients** | 5,102 | 0.12 | 0.24 | 0.18 | 0.18 | 0.07 | 0.21 |  |  |

|  |  | **Genotype frequencies** | | | | | |  |  |
| --- | --- | --- | --- | --- | --- | --- | --- | --- | --- |
|  | **Number of individuals** | **ALA6/ALA7** | **ALA6/ALA5** | **ALA7/ALA5** | **ALA7/ALA7** | **ALA6/ALA6** | **ALA5/ALA5** | **χ2** | **p-value** |
| **Female ASD patients** | 1,028 | 0.13 | 0.21 | 0.18 | 0.19 | 0.07 | 0.22 | 5.67 | 0.340 |
| **ASD patients** | 5,102 | 0.12 | 0.24 | 0.18 | 0.18 | 0.07 | 0.21 |  |  |

|  | **number of alleles transmitted** | **number of alleles not transmitted** | **χ2** | **p-value** |
| --- | --- | --- | --- | --- |
| **ALA5** | 30 | 22 | 1.23 | 0.26 |
| **ALA6** | 23 | 24 | 0.02 | 0.88 |
| **ALA7** | 15 | 22 | 1.32 | 0.25 |

**Table S5.** Transmitted and not transmitted GPx1 alleles and related statistics in unaffected trios.
